# Ultraviolet photodetectors based on ZnO nanorods-seed layer effect and metal oxide modifying layer effect

**DOI:** 10.1186/1556-276X-6-147

**Published:** 2011-02-15

**Authors:** Hai Zhou, Guojia Fang, Nishuang Liu, Xingzhong Zhao

**Affiliations:** 1 Department of Electronic Science and Technology and Key Laboratory of Artificial Micro- and Nano-structures of Ministry of Education, School of Physics and Technology, Wuhan University, Wuhan 430072, People's Republic of China

## Abstract

Pt/ZnO nanorod (NR) and Pt/modified ZnO NR Schottky barrier ultraviolet (UV) photodetectors (PDs) were prepared with different seed layers and metal oxide modifying layer materials. In this paper, we discussed the effect of metal oxide modifying layer on the performance of UV PDs pre- and post-deposition annealing at 300°C, respectively. For Schottky barrier UV PDs with different seed layers, the MgZnO seed layer-PDs without metal oxide coating showed bigger responsivity and larger detectivity (*D*_λ_*) than those of PDs with ZnO seed layer, and the reason was illustrated through energy band theory and the electron transport mechanism. Also the ratio of *D*_254_* to *D*_546_* was calculated above 8 × 10^2 ^for all PDs, which demonstrated that our PDs showed high selectivity for detecting UV light with less influence of light with long wavelength.

## Introduction

Recently, a one-dimensional (1D) nanomaterial has attracted a lot of attention both for fundamental research and potential nano-device applications because of its peculiar characteristics and quantum size effect [1,2]. Among the various nano-structured materials, due to their direct and wide energy bandgap (3.37 eV), ZnO nanorods (NRs) are a promising functional material as potential candidates for short-wavelength optoelectronics applications such as nanoscale lasers [3], light-emitting diodes [4], and ultraviolet (UV) photodetectors (PDs) [5-9]. Although ZnO has many advantages, the existence of many defects of ZnO NRs prepared by hydrothermal method [10] may benefit the formation of ohmic contacts at the electrode/ZnO NRs interface, which is an obstacle to applications in PDs due to its slow response and recovery behaviors.

The Schottky barrier plays an important role in improving the performance of the PDs, and many researchers have investigated the Schottky contact between ZnO NRs and metal [11-15], but investigations on effects of metal oxide coating and seed layer on ZnO NW Schottky PDs using post-deposition thermal annealing treatment are scarce. In this study, to investigate the effect of the seed layer and oxide material on the performance of PDs, a simple route to gain Schottky barrier by deposition of Pt electrodes on the top of different oxide material-coated *n*-ZnO NRs, which are prepared by hydrothermal process on different seed layers is introduced. Then, the samples are treated by thermal annealing process to form Schottky contacts. In this article, the authors have discussed the effects of metal oxide-modified layer on the performance of UV PDs pre- and after post-deposition annealing at 300°C. The investigation of PDs with different seed layers shows that the MgZnO seed layer-PDs without metal oxide coating demonstrates bigger responsivity and larger detectivity than those of PDs with ZnO seed layer, and the reason has been illustrated through energy band theory and the electron transport mechanism. Also the ratio of detectivity (*D*_λ_*, *D*_254_* to *D*_546_*) is calculated above 8 × 10^2 ^for all PDs, which demonstrates that our PDs show high selectivity for detecting UV light with lesser influence of light with long wavelength. The attractiveness of this study is the simplicity of the fabrication process, which could easily be scaled up, and our results may pave the way for the application of low-cost ZnO NRs UV PDs.

## Experimental methods

**Figure 1:**
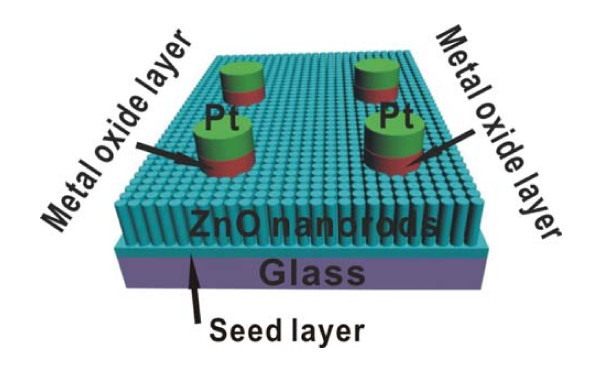
**A schematic diagram of PD**.

The glass substrates were initially cleaned with acetone in an ultrasonic bath, rinsed with deionized water, and then blown dry with dry N_2_. Then, a 120-nm ZnO seed layer was deposited by radio frequency-reactive magnetron sputtering at 100°C. Then, ZnO NRs were grown on ZnO-coated glass substrate by hydrothermal method. The details of the hydrothermal conditions for obtaining ZnO NRs have already been reported elsewhere. In brief, the nutrient solution was an aqueous solution of a 0.05 M zinc nitrate hexahydrate (Zn(NO_3_)_2 _· 6H_2_O) and methenamine (C_6_H_12_N_4_). The reaction was kept at 100°C for 2 h, and then, the ZnO NRs flat film was obtained. Then, to investigate the effect of metal oxide-modified layer on the performance of UV PDs, MgZnO, MgO, and Al-doped ZnO were deposited on ZnO NRs at 100°C by a simple mask plate with radio frequency-reactive magnetron sputtering followed by deposition of 100-nm Pt. The thickness of the metal oxide layer was about 50 nm. Finally, for comparison, a few samples were annealed in air at the temperature of 300°C for 2 h. To investigate the effect of seed layer on the performance of UV PDs, MgZnO seed layer-PDs are prepared without coating oxides, and the experimental conditions were the same as has been mentioned above. A schematic structure of PD with the sample size of 1 × 1 cm^2 ^is shown in the inset of Figure 1, and the photon window area is 1 × 4 mm^2^. The morphology was observed by Sirion field emission scanning electron microscopy (Philips XL30). The photosensitivity was performed using 66984 Xe Arc source (300 W Oriel) and Oriel Cornerstone TM 260 1/4 m Monochromator. The sample was under illumination directly (parallel with the NRs), and the optical power of light was measured by a UV-enhanced Si detector. All the *I*-*V *characteristics were measured using a Keithley 4200 electrometer.

## Results and discussion

**Figure 2:**
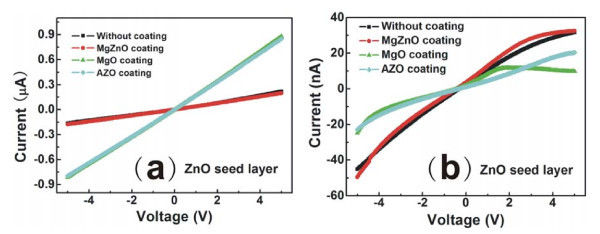
**The *I*-*V *curves of the PDs with different metal oxide coatings**. **(a) **Before annealing; **(b) **after annealing at 300°C.

In our experiment, the as-prepared ZnO NRs grow vertically and closely packed on the ZnO seed layer, the gap between ZnO nanowires is very little, and the average diameter and length of these ZnO NRs are around 90-150 nm and 1.4 μm. The *I*-*V *curves of the PDs with ZnO seed layer are shown in Figure 2a. From the curves, the plots of *I *vs. *V *are straight lines for ZnO seed layer, showing that the contacts at the Pt/ZnO NRs or the Pt/metal oxide interfaces are ohmic. Figure 2b shows the *I*-*V *curves of the PDs annealed at 300°C with ZnO seed layer. It can be seen that when the PDs are annealed at 300°C, the Schottky contacts are obtained. Also, after the annealing process, the dark current of PDs decreases greatly. It has been reported that the as-grown ZnO NRs have large defect concentration, which can be improved by thermal annealing [10]. The authors think that the contacts at the electrode/as-prepared ZnO NRs interface are normally ohmic, which is due to the existence of many defects, such as oxygen vacancies or zinc interstitials, resulting in high carrier density, so that the formation of Schottky contacts is very hard, even with the contacts between as-prepared ZnO NRs and electrodes with high work function metals, such as Au, Ni, and Pt. When ZnO NRs are annealed at certain temperatures, the defects will be reduced, and the defect-related carrier density will also decrease, so that the Schottky contact barrier will be formed.

**Figure 3:**
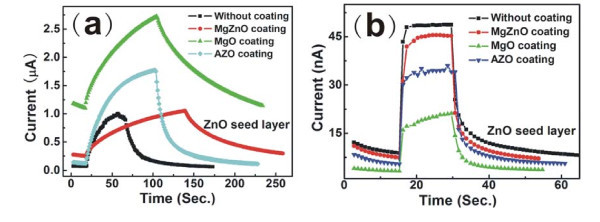
**The dependences of photocurrents on operating time for PDs with different metal oxide coatings under UV light (365 nm) with power density of 16.7 μW/cm^2 ^at the bias of 2 V**. **(a) **Before annealing; **(b) **after annealing at 300°C.

For PDs, the response and recovery times are a very important factor for applications. The dependences of photocurrent on operating time for the PDs with different oxides under UV light (365 nm) with power density of 16.7 μW/cm^2 ^at the bias of 2 V are shown in Figure 3a (before annealing) and Figure 3b (annealed at 300°C), respectively. From Figure 3a, under 365-nm UV illumination, the current of the PDs increases very slowly to reach saturation, and at turn off of the UV lamp, the current decreases also slowly. Also it is deduced that the response time of the PDs is all above 30 s, and the recovery time of the PDs (the photocurrent decreases 80%) is all above 50 s. For the PDs with different oxide-coating materials, the devices show enhanced UV response characteristic, but the response and recovery are all slow. After annealing, all PDs show fast response and recovery behaviors, and their response and recovery times are 3 and 4 s, respectively. For PDs with different oxide coatings, the UV response characteristic gets worse than that of PDs without metal oxide coating, which shows that the effect of metal oxide coating for Schottky contact PDs is a negative one.

It is very well known that the metal oxides deposited at 100°C have some structure defects with high carrier density, which will benefit the formation of ohmic contacts and electron transport. Hence, the metal oxide, as an electron transport layer in PDs, can improve the contact resistance between metal and semiconductor. Therefore, the PDs with metal oxide coating can enhance photoresponse characteristic before annealing. After annealing, the structure defects decrease, and the electrical resistivity of all metal oxides will increase, the photogenerated electrons will be blocked, and very few can be collected by Pt electrode at forward bias. However, for PDs without oxide coatings, the contacts at Pt/ZnO NRs interfaces are improved by annealing process, and the photogenerated electrons can easily reach to Pt electrode at forward bias and get high photocurrent, and PDs without oxide coatings show fast response and recovery behavior after annealing. Therefore, it is concluded that the PDs without oxide coatings display better performance than those with oxide coatings.

**Figure 4:**
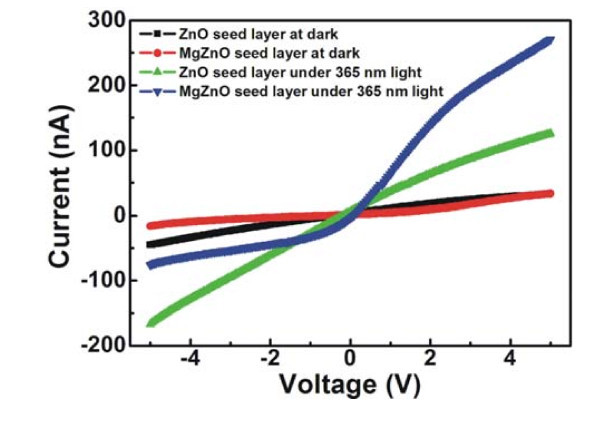
**The *I*-*V *curves of the PDs based on ZnO or MgZnO seed layer without metal oxide coating measured at dark and under 365 nm UV light**.

In order to investigate the effect of the seed layer on the performance of PDs, ZnO NRs are prepared with two kinds of seed layers (MgZnO and ZnO seed layers). Herein, high pure ZnO is chosen for the matching of energy band with that of ZnO NRs. MgZnO is chosen due to its low carrier density and large band gap (about 4.0 eV). Figure 4 shows the *I*-*V *curves of the PDs without oxide coating and annealed at 300°C, which demonstrates the electron transport characteristics of PDs with different seed layers at dark and under 365-nm UV light, respectively. From the curves, it can be seen that the contacts between Pt and ZnO NRs are good Schottky contacts. At dark, the PD with MgZnO seed layer has lower dark current than that with ZnO seed layer, which may be attributed to the lower carrier density of MgZnO film. Under 365-nm UV light, the current of the PD with MgZnO seed layer is higher than that of the PD with ZnO seed layer at forward bias. The ratios of photocurrent to dark current $\left(R_{I_{\text{Ph}}/I_{\text{D}}}\right)$ calculated for the PDs with MgZnO and ZnO seed layer at 5 V are 3.9 and 8.2, respectively.

For Schottky barrier PDs, the actual barrier height at the electrode/semiconductor interface is an important part of the PDs under investigation. The Schottky barrier height can be determined using *I*-*V *measurements as per Equation (1) [13]

(1) $$I=\left[A^{*}AT^{2}\exp\left(-\frac{q\Phi_{\text{B}}}{KT}\right)\right]\left[\exp\left(\frac{qV}{nKT}\right)-1\right]$$

where *n *is the ideal factor, *K *is the Boltzmann's constant, *T *is the absolute temperature, Φ_B _is the barrier height, *A *is the Schottky contact area, and A* is the effective Richardson coefficient constant. By means of forward biased *I*-*V *measurements and Equation (1), it can be deduced that for the PDs with ZnO and MgZnO seed layer, Schottky barrier heights Φ_B _at the Pt/ZnO NRs interface are, respectively, about 0.768 and 0.796 eV at dark and the respective Φ_B _values are about 0.738 and 0.734 eV under 365-nm light. From above, it can be seen that Φ_B _decreases under 365-nm light, and it decreases by 0.03 and 0.062 eV for the PDs with ZnO and MgZnO seed layer, respectively. The decrease of Φ_B _for the PDs with MgZnO seed layer is two times that for the PDs with ZnO seed layer, which illustrates that the larger increase of photocurrent will result in the larger decrease of Φ_B_.

**Figure 5:**
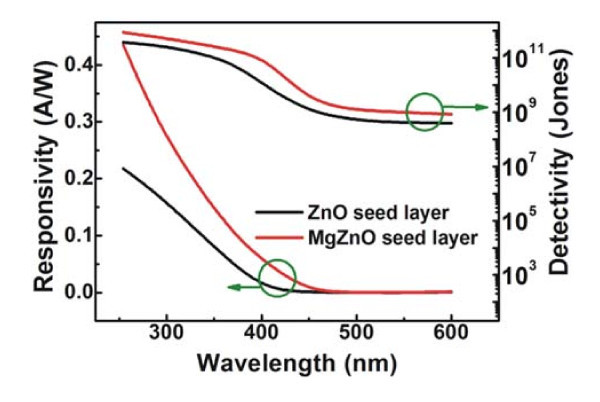
**The spectral responsivity and detectivity curves of PDs without metal oxide coating under the forward biases of 2 V**.

The responsivity (*R*) is an important parameter to reflect the performance of PDs, and so the spectral *R *curves obtained from non-oxide-coated PDs annealed at 300°C with different seed layer under the forward biases of 2 V are presented in Figure 5. From these spectra, it can be seen that the responsivity of the PDs with MgZnO seed layer is higher than that of the PDs with ZnO seed layer and reaches to as high as 0.44 A/W at 254 nm, which is double that of PDs with ZnO seed layer (0.22 A/W). The detectivity is also calculated, which is given by the following [16]:

(2) $$D^{*}=\frac{R}{{\left(2qJ_{\text{d}}\right)}^{\frac{1}{2}}}=\frac{J_{\text{ph}}}{L_{\text{light}}}\frac{1}{{\left(2qJ_{\text{d}}\right)}^{\frac{1}{2}}}$$

where *R *is the responsivity of the photodiode, *J*_d _is the dark current, *J*_ph _is the photocurrent density, and *L*_light _is the light intensity. Detectivity is calculated and also plotted in Figure 5. From the curves of the detectivity of PDs, it can be noted that the Schottky barrier PDs exhibited spectral response mainly in the range from 250 to 400 nm, with the detectivity above 10^11 ^Jones (1 Jones = 1 cmHz^1/2^/W), and the detectivity of the PDs with MgZnO seed layer is higher than that of the PDs with ZnO seed layer. At the wavelength above 400 nm, the PDs show little detectivity, and the detectivity decreases with the increase of the wavelength. The ratio of *D*_254_* to *D*_546_* is above 8 × 10^2^, which shows that the PDs have high selectivity for detecting UV light with less influence of light with long wavelength.

**Figure 6:**
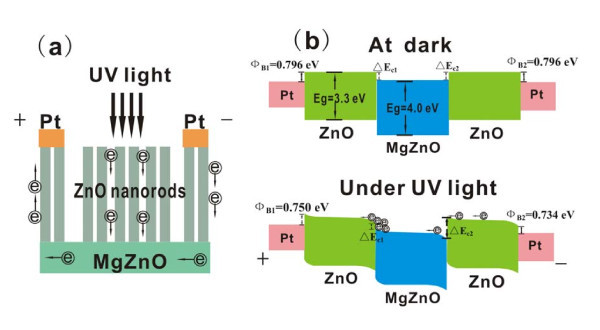
**Carrier transport processes in the ZnO NRs PDs**. **(a) **Photogenerated electron transport route under UV illumination. **(b) **Schematic energy level diagrams of the PD with MgZnO seed layer at dark and under UV illumination, respectively.

In order to explore the enhanced performance of PDs with MgZnO seed layer, carrier transport processes in the ZnO NRs PDs under forward bias are illustrated in Figure 6a. In the dark, oxygen is adsorbed at the surface of the NRs to form a chemically adsorbed surface state. Under UV illumination, electron-hole pairs are generated when photon energy exceeds the energy band gap (*hυ *>*E*_g_). Photogenerated holes move to the surface of ZnO NRs and the adsorbed oxygen is photodesorbed, and unpaired electrons in the NRs migrate to the electrodes at a bias voltage and contribute to the photocurrent [6,12]. From Figure 6a, it can be seen that the photogenerated electrons, generated from the surface of ZnO NRs, move to the MgZnO layer at first, and then move from MgZnO to ZnO NRs, which are underneath the electrode, and finally reach to the electrode. Owing to the high contact resistance among NRs, a few photogenerated electrons may pass from NRs and contribute to the photocurrent. In Figure 6b, the Schottky barrier height Φ_B _is calculated using forward- or reverse-biased *I*-*V *measurements and Equation (1). From Figure 6b, it can be seen that at dark, the barrier height between ZnO NRs and MgZnO (Δ*E*_c1_) is the same as that between MgZnO and ZnO NRs (Δ*E*_c2_). Under UV illumination, Δ*E*_c2 _gets larger, and Δ*E*_c1 _gets smaller at forward bias, which benefits the photogenerated electrons moving from ZnO NRs to MgZnO. Owing to existence of the small Δ*E*_c1_, the photogenerated electrons will collect together at the ZnO NRs/MgZnO interface, and then the two-dimensional electron gas (2DEG) will form [17]. The 2DEG will decrease the transverse resistances between the interface strongly [18], and then the photogenerated electrons may reach easily to Pt electrode. Therefore, compared with the PDs with ZnO seed layer, the PDs with MgZnO seed layer can realize bigger responsivity and higher detectivity.

## Conclusions

In conclusion, Schottky barrier PDs based on ZnO NRs were prepared by varying seed layers and metal oxide-coating materials. Before annealing, PDs coated with metal oxide materials showed enhanced photoresponse compared to that without coatings. However, after annealing treatment, the metal oxides will block photogenerated electrons to electrodes and reduce photocurrent. Also, after annealing at 300°C, contacts at the electrode/ZnO NRs or electrode/oxide interface were Schottky type, and the performance of the PDs has improved with the great decrease of response and recovery times. For different seed layer-PDs without oxide coating, the PDs with MgZnO seed layer showed bigger responsivity and lager detectivity than those of PDs with ZnO seed layer, and the ratio of *D*_254_* to *D*_546_* was above 8 × 10^2 ^for all PDs. The results may provide a simple route to obtain low-cost high performance UV PDs.

## Abbreviations

NR: nanorod; PDs: photodetectors; UV: ultraviolet.

## Competing interests

The authors declare that they have no competing interests.

## Authors' contributions

All authors contributed equally and read and approved the final manuscript.
